# TROP-2, Nectin-4, GPNMB, and B7-H3 Are Potentially Therapeutic Targets for Anaplastic Thyroid Carcinoma

**DOI:** 10.3390/cancers14030579

**Published:** 2022-01-24

**Authors:** Soji Toda, Shinya Sato, Nao Saito, Kazumasa Sekihara, Ai Matsui, Daisuke Murayama, Hirotaka Nakayama, Nobuyasu Suganuma, Yoichiro Okubo, Hiroyuki Hayashi, Hiroyuki Iwasaki, Yohei Miyagi, Daisuke Hoshino

**Affiliations:** 1 Department of Breast and Endocrine Surgery, Kanagawa Cancer Center, Yokohama 241-8515, Japan; s-toda@kcch.jp (S.T.); a-matsui@kcch.jp (A.M.); d-murayama@kcch.jp (D.M.); iwasaki.h@kcch.jp (H.I.); 2 Molecular Pathology and Genetics Division, Kanagawa Cancer Center Research Institute, Yokohama 241-8515, Japan; ssato53@gancen.asahi.yokohama.jp (S.S.); miyagi@gancen.asahi.yokohama.jp (Y.M.); 3 Department of Cancer Biology, Kanagawa Cancer Center Research Institute, Yokohama 241-8515, Japan; nasaito@gancen.asahi.yokohama.jp (N.S.); sekihara@gancen.asahi.yokohama.jp (K.S.); 4 Department of Surgery, Hiratsuka Kyosai Hospital, Hiratsuka 254-8502, Japan; nakayama-h@kkr.hiratsuka.kanagawa.jp; 5 Department of Surgery, Yokohama City University, Yokohama 236-0004, Japan; suganuma@yokohama-cu.ac.jp; 6 Department of Pathology, Kanagawa Cancer Center, Yokohama 241-8515, Japan; yoichiro0207@hotmail.com; 7 Department of Pathology, Yokohama Municipal Citizen’s Hospital, Yokohama 221-0855, Japan; hirohayam@yahoo.co.jp

**Keywords:** ADC, ATC, *HER2*, TROP-2, Nectin-4, GPNMB, B7-H3

## Abstract

**Simple Summary:**

Anaplastic thyroid carcinoma is a highly aggressive thyroid tumor with a poor prognosis. There are limited choices for the effective treatment of this type of carcinoma. Whether the targets of antibody–drug conjugates are expressed in anaplastic thyroid carcinoma remains unclear. Therefore, we examined expression rates of the following antibody–drug conjugate targets using the tissue microarrays of anaplastic thyroid carcinomas: *human epidermal growth factor receptor 2*, *nectin-4*, *trophoblast cell surface antigen 2*, *glycoprotein non-metastatic B*, and *B7-H3*. We found that *glycoprotein non-metastatic B* and *B7-H3* were expressed in most anaplastic thyroid carcinoma tissues. *Trophoblast cell surface antigen 2* and *nectin-4* were expressed in 65% and 59% of anaplastic thyroid carcinoma tissues, respectively. *Trophoblast cell surface antigen 2* was high expressed in anaplastic thyroid carcinoma undifferentiated from papillary thyroid carcinoma. In contrast, *nectin-4* expression was high in patients with de novo anaplastic thyroid carcinoma. These cell membrane proteins are potential therapeutic targets for anaplastic thyroid carcinoma.

**Abstract:**

Background: Anaplastic thyroid carcinoma (ATC) is a highly aggressive thyroid tumor with a poor prognosis. However, there are limited choices for ATC treatment. Recently, the effectiveness of antibody–drug conjugates has been demonstrated in various carcinomas. Whether the targets of antibody–drug conjugates are expressed in anaplastic thyroid carcinoma remains unclear. Methods: Fifty-four patients with ATC were enrolled in this study. Tissue microarrays were constructed using the archives of formalin-fixed paraffin-embedded tissue blocks. All sections were stained with the following antibody–drug conjugate targets: *human epidermal growth factor receptor 2* (*HER2*), *nectin-4*, *trophoblast cell surface antigen 2* (*TROP-2*), *glycoprotein non-metastatic B* (*GPNMB*), and *B7-H3*. Results: *HER2* was negative in all tissues, whereas *GPNMB* and *B7-H3* were expressed in most ATC tissues. *TROP-2* and *nectin-4* were expressed in 65% and 59% of ATC tissues, respectively. *TROP-2* was expressed at significantly higher levels in ATC undifferentiated from papillary thyroid carcinoma than in ATC undifferentiated from follicular thyroid carcinoma and de novo ATC. In contrast, *nectin-4* expression was markedly higher in patients with de novo ATC than in those with papillary and follicular thyroid carcinoma. Conclusions: *TROP-2* and *nectin-4* are potential therapeutic targets for ATC undifferentiated from papillary thyroid carcinoma and de novo ATC, respectively. *GPNMB* and *B7-H3* potential for treating all types of ATC.

## 1. Introduction

Although it is a rare form of thyroid cancer, accounting for approximately 1–2% of all thyroid cancers, anaplastic thyroid carcinoma (ATC) is a particularly aggressive type of carcinoma. The median survival time of patients with ATC is only 3–6 months after diagnosis [1].

Primary ATC is an ATC without a pre-existing history of differentiated thyroid carcinoma (DTC). Secondary ATC is an ATC with a DTC history or coexisting DTC components at the time of diagnosis. DTC converted to ATC has been confirmed by the next-generation sequencing of cancer-associated gene panels using serial biopsies [2,3,4]. There are four genetically distinct types of ATC: *v-RAF murine sarcoma viral oncogene homolog B* (*BRAF*) mutation cluster, *neuroblastoma RAS viral oncogene homolog* mutation cluster, *phosphatase and tensin homolog/neurofibromin 1/RB transcriptional corepressor 1* mutation cluster, and loss-of-function in the *CDKN2A* and *CDKN2B* clusters. These clusters have the genetic features of papillary carcinoma or follicular carcinoma, supporting the hypothesis of anaplastic transformation through the acquisition of additional oncogenic alterations [5].

Recently, *BRAF* and *mitogen-activated protein kinase kinase* (*MEK*) inhibitors have shown a high response rate in *BRAF* V600E-mutated ATC [6]. However, we still have no other practical option for treating ATC, regardless of *BRAF* mutation status.

Antibody–drug conjugates (ADCs) are compound-binding antibodies with cytotoxic low-molecular-weight drugs via chemical linkers; they expand the repertoire of oncology therapeutics. ADCs bind to the antigen on the cell membrane surface and are internalized by endocytosis. The linker is then cleaved and releases the drug into the target tumor cells. Gemtuzumab ozogamicin, which has an antibody against the cluster of differentiation (CD) 33, was the first ADC approved for acute myeloid leukemia. In solid cancer, ADCs targeting *human epidermal growth factor receptor 2* (*HER2*), such as trastuzumab emtansine and trastuzumab deruxtecan, showed favorable outcomes in *HER2*-positive breast or gastric cancer [7,8,9].

In a recent study, sacituzumab govitecan, an ADC comprising an antibody targeting the human *trophoblast cell surface antigen 2* (*TROP-2*), extended progression-free and overall survival compared to single-agent chemotherapy among patients with metastatic triple-negative breast cancer [10]. Enfortumab vedotin, an ADC directed against *nectin-4*, prolonged patients’ survival compared to standard chemotherapy treatments in patients with urothelial carcinoma [11]. In addition, some clinical trials of ADCs targeting proteins on cell membranes, such as *glycoprotein non-metastatic B* (*GPNMB*) and *B7-H3*, are currently in progress [12,13]. *TROP-2* was detected in 50% of 24 ATC cases [14], and *HER2* expression has been rarely observed in ATC cases [15,16]. However, no studies have evaluated the expression of other ADC target proteins, such as *nectin-4*, *GPNMB*, and *B7-H3* in ATCs.

In this study, we evaluated the expression levels of *HER2*, *TROP-2*, *nectin-4*, *GPNMB*, and *B7-H3* by immunostaining in ATC patient tissues. We selected ADC targeting proteins whose clinical trials are progressing in Phase I/II or higher. We found that the expression of the proteins differs depending on the pathological types of ATC progression and that ADCs are viable candidates for ATC treatment.

## 2. Materials and Methods

### 2.1. Patients and Samples

A total of 54 patients with ATC who were treated at Kanagawa Cancer Center from 1990 to 2009 were enrolled in this study. The study design was approved by the Ethics Committee of the Kanagawa Cancer Center (approval number, 19–34).

Stage classification was performed according to the 8th edition of the tumor-node-metastasis classification system of the American Joint Committee on Cancer (AJCC) and the Union for International Cancer Control. Pathological types were divided into three types based on the origin of the tumor: papillary thyroid carcinoma (PTC), follicular thyroid carcinoma (FTC), and de novo ATC. ATC coexisting with PTC was defined as PTC-origin type, ATC coexisting with FTC as FTC-origin type, and ATC where DTC does not coexist as de novo type.

Tissue microarrays (TMAs) were constructed using formalin-fixed paraffin-embedded tissue blocks. Three cores of tumor tissue and a core of non-neoplastic thyroid tissue, if available, were selected, and the designed tumor zone on each block was punched in a 4 mm diameter circle. TMA blocks were cut into 4 μm thick sections and subjected to the usual immunohistochemistry protocol. In this study, we evaluated the cores that contained ATC tissue. All sections were stained with *HER2* (mouse monoclonal antibody, Nichirei, Tokyo, Japan, 413431, 1/50 dilution), *nectin-4* (rabbit monoclonal antibody, Abcam, Cambridge, UK, ab192033, 1/3000 dilution), *TROP-2* (mouse monoclonal antibody, Santa Cruz Biotechnology, Inc., Dallas, TX, USA, sc376181, 1/50 dilution), *GPNMB* (mouse monoclonal antibody, Proteintech, Rosemont, IL, USA, 66926, 1/1000 dilution), and *B7-H3* (mouse monoclonal antibody, Proteintech, Rosemont, IL, USA, 66481, 1/2500 dilution). The exposed antigen was detected using Histofine simple stain MAX PO (Nichirei, Tokyo, Japan). The reaction was identified with DAB (Nichirei, Tokyo, Japan) and counterstained with Mayer’s hematoxylin (FUJiFILM, Osaka, Japan)

### 2.2. Evaluation of Immunohistochemical Staining

**Figure 1:**
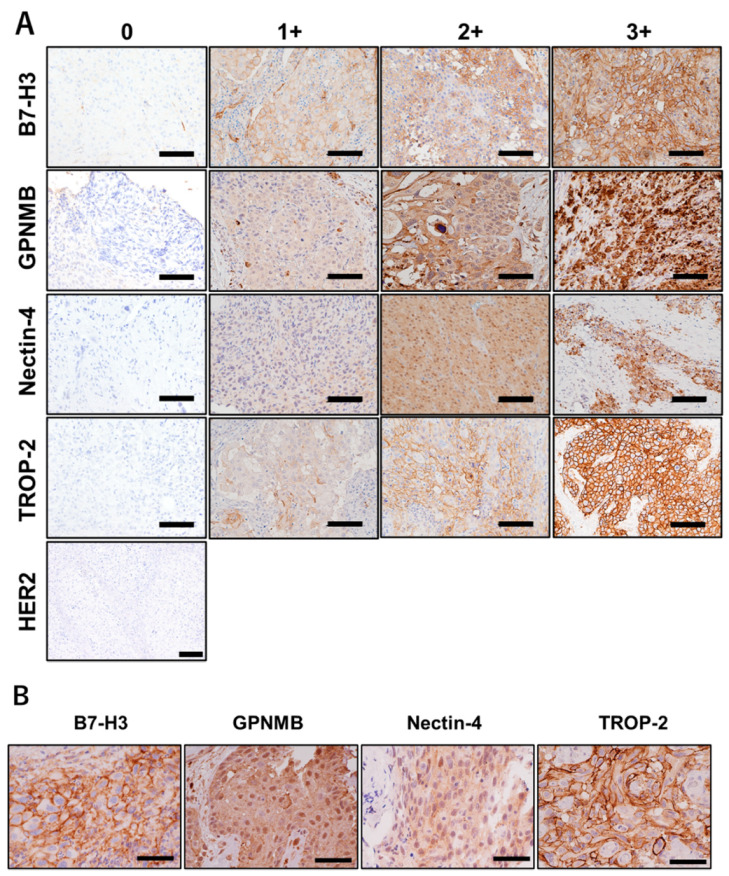
The immunohistochemistry staining examples of *human epidermal growth factor receptor 2 (HER2)*, *trophoblast cell surface antigen 2*, *nectin-4*, *glycoprotein non-metastatic B*, and *B7-H3*. Staining intensity was scored as 0 (negative), 1+ (weakly positive), 2+ (moderate), and 3+ (strongly positive). *HER2* expression was negative in all anaplastic thyroid carcinoma samples ((**A**) scale bar represents 100 μm). The higher magnification showed that *TROP-2* and *B7-H3* were strongly stained on the cell membrane compared to *nectin-4* and *GPNMB* ((**B**) scale bar represents 50 μm).

Evaluation of immunohistochemistry was performed based on the Allred score [17,18,19,20,21,22]. We graded the intensity score (IS) to classify the specimens into four grades as follows: 0, negative; 1, weak; 2, moderate; and 3, strong (Figure 1). Next, the proportion score (PS) was determined as follows: 0, 0%; 1, <1%; 2, 1–10%; 3, 11–33%; 4, 34–66%; and 5, 67–100%. The total score (TS) was calculated as the sum of IS and PS. We assigned a rating of positive when the TS was 3 or higher. Immunohistochemical scoring was evaluated by an experienced pathologist who was blinded to the clinical parameters.

### 2.3. Statistical Analysis

Statistical analysis was performed using EZR (Saitama Medical Center, Jichi Medical University, Saitama, Japan), which is a graphical user interface for R (R Foundation for Statistical Computing, Vienna, Austria). The Fisher’s exact test was employed to evaluate the correlations with the clinical characteristics. Overall survival was tested using log-rank tests. The distribution of time-to-event endpoints for overall survival was estimated using the Kaplan–Meier method. The TS of *TROP-2* and *nectin-4* were compared among the three groups (PTC-origin type, FTC-origin type, and de novo type) using Kruskal–Wallis/Steel–Dwass tests. An outcome of *p* < 0.05 was considered to be statistically significant.

## 3. Results

### 3.1. Patient Characteristics

A total of 54 patients with ATC who were treated at the Kanagawa Cancer Center between 1990 and 2009 were enrolled in this study. The patients had a median age of 69 (range, 47–90) years; the clinical stages classified by the AJCC staging system 8th edition included stage IVA (5.5%), IVB (55.5%), and IVC (39%). The prognostic index (PI) is based on the number of unfavorable characteristics, including acute symptoms, large tumors, distant metastasis, and leukocytosis [23]. In all, 38% of the patients had a PI score of 0 or 1, and 62% had a PI score of 2, 3, or 4. The median overall survival time was 176 days, and the number of patients who survived at 3 years was only five. The pathological types divided by the origin of the tumor included the PTC-origin type (37%), FTC-origin type (17%), and de novo type (46%). Detailed patient characteristics are summarized in Table 1.

### 3.2. Distribution of the Immunohistochemical Total Score

**Figure 2:**
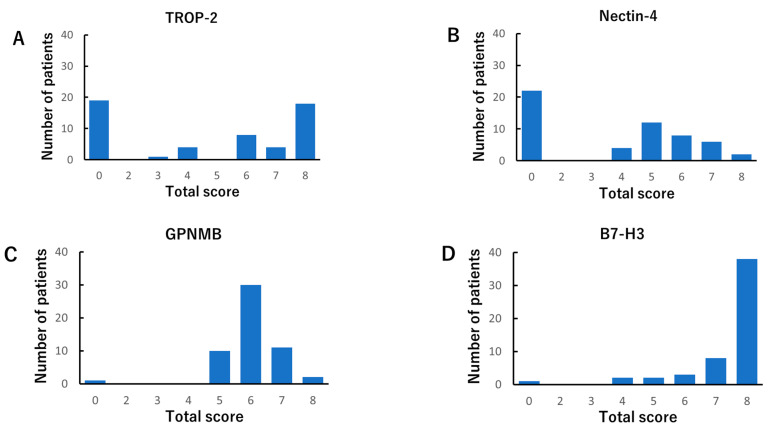
The immunohistochemistry total score distributions of *trophoblast cell surface antigen 2* (*TROP-2*) (**A**), *nectin-4* (**B**), *glycoprotein non-metastatic B* (*GPNMB*) (**C**), and *B7-H3* (**D**) in anaplastic thyroid carcinoma tissues. We rated them positive when the total score was 3 or higher. Each percentage of positive in the total score was as follows: *HER2* (0%), *TROP-2* (65%), *nectin-4* (59%), *GPNMB* (98%), and *B7-H3* (98%).

**Figure 3:**
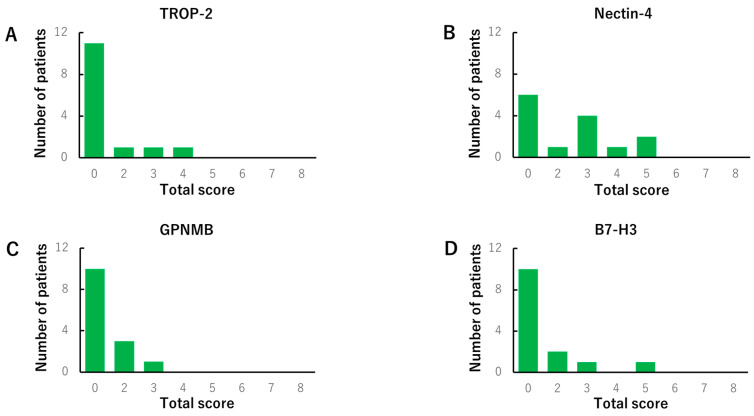

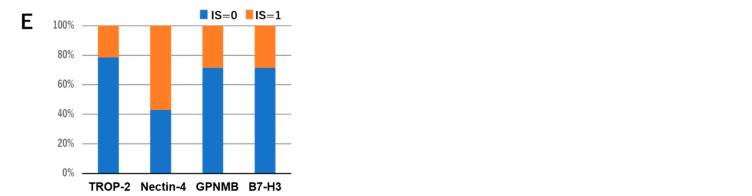
The immunohistochemistry total score distribution of *trophoblast cell surface antigen 2* (*TROP-2*) (**A**), *nectin-4* (**B**), *glycoprotein non-metastatic B* (*GPNMB*) (**C**), and *B7-H3* (**D**) in normal thyroid tissues. We rated them positive when the total score was 3 or higher. Each percentage of positive in the total score was as follows: *TROP-2* (14%), *nectin-4* (50%), *GPNMB* (7%), and *B7-H3* (14%). No moderate or strong staining, whose intensity score (IS) was 2 or 3, was observed in all normal tissues (**E**).

We evaluated the TS of *HER2*, *TROP-2, nectin-4, GPNMB*, and *B7-H3* using the IS and PS, as described in the Methods section. *HER2* was found to be negative in all tissues (Figure 1A), as previously reported [15,16]. *TROP-2* and *B7-H3* were predominantly stained on the cell membrane, while *nectin-4* and *GPNMB* were stained in the cytoplasm (Figure 1B). Both *GPNMB* and *B7-H3* were expressed in most ATC tissues. In contrast, both *TROP-2* and *nectin-4* were expressed in 65% and 59% of ATC tissues, respectively (Figure 2). In normal thyroid tissues, the TS-positive rates of *TROP-2*, *nectin-4*, *GPNMB*, and *B7-H3* were 14%, 50%, 7%, and 14%, respectively. No moderate or strong staining with an IS of 2 or 3 was observed in all normal tissues (Figure 3).

### 3.3. The Total Score of Human Trophoblast Cell Surface Antigen 2 and nectin-4 Is Associated with Histological Types

**Figure 4:**
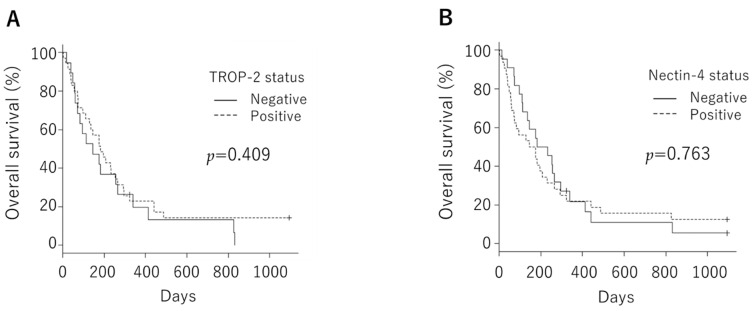
Overall survival was not significantly different between the negative and positive groups, neither in *trophoblast cell surface antigen 2* (median, 146 days vs. 183 days; *p* = 0.409 (**A**)) nor in *nectin-4* (median, 207.5 days vs. 159.0 days; *p* = 0.763 (**B**)).

**Figure 5:**
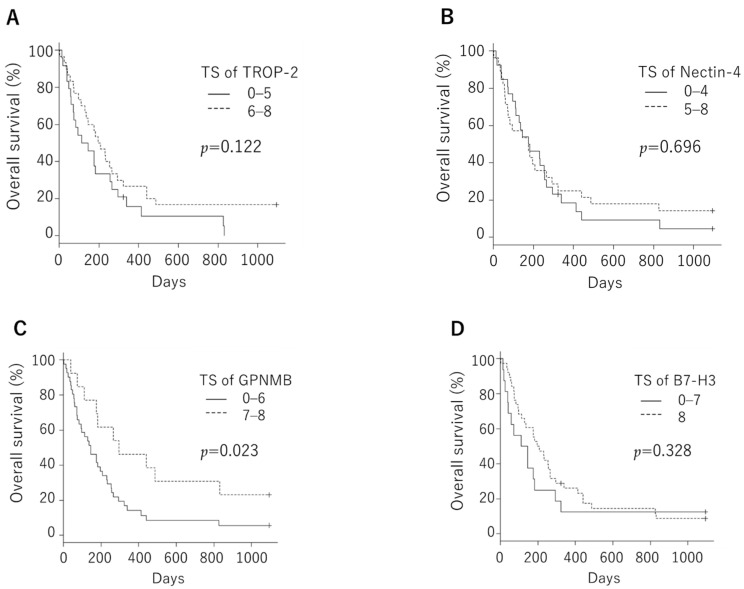
Overall survival was not significantly different between the negative and positive groups, neither in *trophoblast cell surface antigen 2* even when the cutoff was set to median total score (median, 129.0 days vs. 201.5 days; *p* = 0.122 (**A**)) nor in *nectin-4* (median, 179.5 days vs. 174.5 days; *p* = 0.696 (**B**)). Patients with low *GPNMB* expression had significantly poorer prognoses than those with high expression when the cutoff was set to median total score (median, 144 days vs. 296 days; *p* = 0.0231, (**C**)), while there was no significant difference in *B7-H3* expression (median, 127 days vs. 201.5 days; *p* = 0.328 (**D**)).

**Figure 6:**
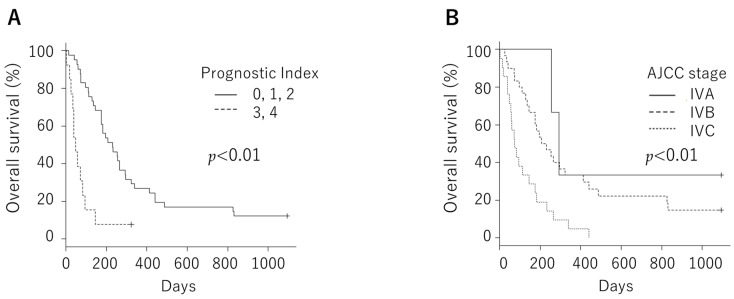
Overall survival was significantly different in known prognostic factors such as the prognostic index (median, 231 days vs. 49 days; *p* < 0.01 (**A**)) or the AJCC stage (median, 293 days vs. 219 days vs. 74 days; *p* < 0.01 (**B**)).

We tested whether *TROP-2* and *nectin-4* expressions were associated with the clinical course. The overall survival rate was not significantly different between the negative and positive groups, neither in *TROP-2* (median, 146 days vs. 183 days; *p* = 0.409) nor in *nectin-4* (median, 207.5 days vs. 159.0 days; *p* = 0.763, Figure 4). We examined this cohort in a similar manner with a median TS cutoff, but there was also no significant difference. Overall survival was examined with a median TS cutoff in *GPNMB* and *B7-H3*. Patients with low *GPNMB* expression had significantly poorer prognoses than those with high expression (median, 144 days vs. 296 days; *p* = 0.0231, Figure 5). However, it was confirmed that there was a significant difference in overall survival rates at the AJCC stage and the prognostic index, which are known prognostic factors (Figure 6). Next, we evaluated the correlations of the *TROP-2*, nectin-4, *GPNMB*, and *B7-H3* expressions with various clinical factors. The AJCC Stage in *TROP-2* and the rapid growth in nectin-4 showed a significant difference (Table 2). 

**Figure 7:**
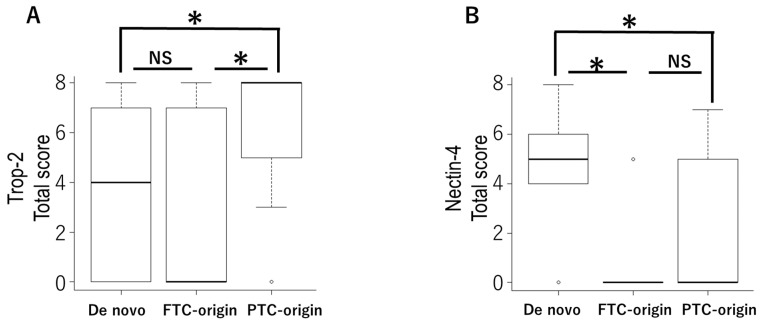

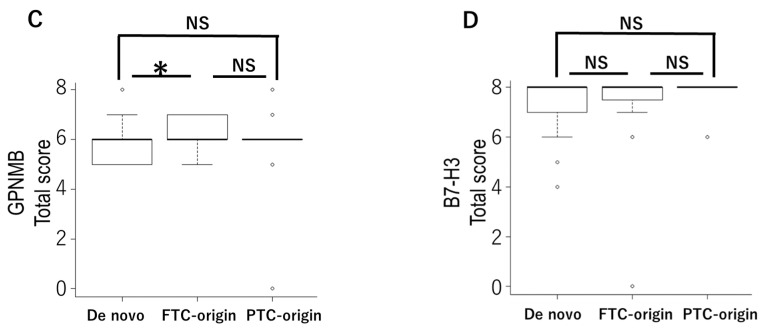
Pathological types are related to the expression of *trophoblast cell surface antigen 2* (*TROP-2*) and *nectin-4*. The total score of *TROP-2* was significantly higher in the papillary thyroid carcinoma (PTC)-origin type group than the follicular thyroid carcinoma (FTC)-origin type and de novo type groups (**A**). The total score of *nectin-4* was significantly higher in the de novo type than in the PTC-origin and FTC-origin types (**B**). Although the total score of *GPNMB* was significantly higher in the FTC-origin group than in the de novo group, both had a median total score of 6 (**C**). The total score of *B7-H3* was not different in any histological type (**D**); NS, not significant. * *p* < 0.05 (Kruskal–Wallis test).

Regarding the histological types, the TS of *TROP-2* was significantly higher in the PTC-origin type group than in the FTC-origin type and de novo type. In contrast, the TS of *nectin-4* was markedly higher in the de novo type than in the PTC-origin and FTC-origin types. The TS of *B7-H3* was not different in any histological type. Although the TS of *GPNMB* was significantly higher in the FTC-origin group than in the de novo group, both had a median TS of 6 (Figure 7).

## 4. Discussion

ATC is an undifferentiated tumor of the thyroid follicular epithelium, which is highly aggressive and has a poor prognosis. Despite numerous attempts to explore new therapeutic targets associated with ATC’s prognosis, no effective treatment has been found to date. In the present study, we investigated whether ADC target protein expression in ATC may be a novel treatment for ATC.

We evaluated the correlations of the *TROP-2*, *nectin-4*, *GPNMB*, and *B7-H3* expressions with various clinical factors, but many factors showed no significant differences. Although the AJCC stage in *TROP-2* and the rapid growth in *nectin-4* showed significant differences, there were no significant differences in overall survival rates. The positivity rates of *TROP-2* were 95% in 20 PTC-origin ATC cases, 33% in 9 FTC-origin ATC cases, and 52% in 25 de novo ATC cases. The TS of *TROP-2* was significantly high in the PTC-origin type. In contrast, the *nectin-4* expression rates were 45% in 20 PTC-origin ATC cases, 22% in 9 FTC-origin ATC cases, and 84% in 25 de novo ATC cases. The TS of *nectin-4* was significantly high in de novo ATC cases. Thus, *TROP-2* and *nectin-4* expressions are related to the histological types. These results suggest that *TROP-2*-targeted therapy is expected in PTC-origin ATC cases, and *nectin-4* targeted therapy is expected in de novo ATC cases. While the TS of *B7-H3* was not different in any histological types, the TS of *GPNMB* was significantly higher in the FTC-origin group than in the de novo group. Since both had a median TS of 6, this has little clinical significance.

When evaluating the expression of cell membrane proteins by immunohistochemistry staining, each report has a different standard for positivity. We defined expressions as positive when the TS was 3 or higher in order to detect ATCs which express the target proteins and are candidates for ADCs. We showed that there was no significant difference in overall survival levels between *TROP2* and *nectin-4* when the cutoff of the TS is defined as 3 or higher. However, there was also no significant difference in overall survival with a median TS cutoff. Overall survival was examined with a median TS cutoff in *GPNMB* and B7-H3. Patients with a low *GPNMB* expression had significantly poorer prognoses than those with a high expression. Although *GPNMB* could be a prognostic factor, it should be noted that this result is highly influenced by a group whose TS is 6, since this group accounts for 56% of *GPNMB*. In terms of the staining on cell membranes, *TROP-2* and *B7-H3* were strongly stained on cell membranes compared to *nectin-4* and *GPNMB*. Although *TROP-2* and *B7-H3* are the ideal candidates for ADC therapy, further study on the effectiveness of ADC is still needed. *Nectin-4* and *GPNMB* are also potential therapeutic targets because some reports showed that ADCs are effective even when the expression of the target protein is low [8].

We confirmed that *HER2* expression was not observed in 54 patients with ATC, as previously reported [15,16]. In contrast, *B7-H3* and *GPNMB* expressions were positive in 98% of 54 patients with ATC. Thus, *B7-H3* and *GPNMB* are potential therapeutic targets in patients with ATC.

*B7-H3*, also known as *CD276*, is a member of the B7 family of immunomodulatory molecules [24,25]. In addition, *GPNMB*, also referred to as *osteoactivin*, is a transmembrane glycoprotein that is associated with increased invasion and metastasis, decreased apoptosis, and enhanced angiogenesis [26]. Overexpression of *B7-H3* and *GPNMB* is associated with the risk of recurrence and of shorter overall survival [27,28,29,30,31]. *B7-H3* and *GPNMB* are overexpressed in many solid cancers, but their protein expressions are limited in normal tissues [31,32,33,34,35].

The present study also showed immunohistochemistry staining of *TROP-2*, *nectin-4*, *GPNMB*, and *B7-H3* is weaker in normal thyroid tissues than in ATC tissues. This may allow for ADC treatment with limited side effects. In *BRAF* mutant melanomas, *GPNMB* is upregulated after *BRAF/MEK* inhibitor therapy [36]. This suggests that *GPNMB* is a candidate target in *BRAF* mutant ATC after *BRAF/MEK* inhibitor therapy.

In this study, we demonstrated that *TROP-2*-, *nectin-4*-, *B7-H3*-, and *GPNMB*-related ADCs are potential therapeutic options for ATC.

## 5. Conclusions

*TROP-2* and *nectin-4* are potential therapeutic targets for ATC undifferentiated from papillary thyroid carcinoma and de novo ATC, respectively. *GPNMB* and *B7-H3* potential for treating all types of ATC.

## Figures and Tables

**Table 1:** Patient characteristics.

Characteristics		Number of Patients
Sex	Male	27
	Female	27
Age	Median	69 (47–90)
AJCC stage *	IVA	3
	IVB	30
	IVC	21
Tumor size	<50 mm	13
	≥50 mm	41
Rapid growth	Absent	37
	Present	17
White blood cell count	<10,000/mm^3^	37
	≥10,000/mm^3^	17
Surgical curativity	Curative	18
	Non-curative	36
Prognostic index **	0, 1	21
	2, 3, 4	33
Pathological type ***	PTC-origin type	20
	FTC-origin type	9
	De novo type	25

* The American Joint Committee on Cancer tumor-node-metastasis staging system, 8th edition. ** The prognostic index is based on the number of the four unfavorable characteristics the patient possessed: acute symptoms, large tumor (≥50 mm), distant metastasis, and leukocytosis (white blood cell count ≥10,000/mm^3^). *** Pathological types are divided into three types according to the origin of tumor. Anaplastic thyroid carcinoma (ATC) coexisting with papillary thyroid carcinoma (PTC) is defined as PTC-origin type, ATC coexisting with follicular thyroid carcinoma (FTC) as FTC-origin type, and ATC where DTC does not coexist as de novo type.

**Table 2:** Correlation between clinical characteristics and ADC targets expression.

	Number of Patients											
	TROP2			*Nectin-4*			Total Score of *GPNMB*			Total Score of *B7-H3*		
Characteristics	Negative	Positive	*p* Value	Negative	Positive	*p* Value	0–6	7, 8	*p* Value	0–7	8	*p* Value
Gender												
Male	11	16	NS	11	16	NS	21	6	NS	10	17	NS
Female	8	19		11	18		20	7		6	21	
Age												
<70	11	16	NS	9	18	NS	22	5	NS	4	23	0.0352
≥70	8	19		13	14		19	8		12	15	
AJCC Stage *												
ⅣA,B	7	26	<0.01	16	17	NS	24	9	NS	9	24	NS
ⅣC	12	9		6	15		17	4		7	14	
Tumor size												
<50 mm	4	9	NS	8	5	NS	9	4	NS	3	10	NS
≥50 mm	15	26		14	27		32	9		13	28	
Rapid growth												
Absent	15	22	NS	20	17	<0.01	27	10	NS	11	26	NS
Present	4	13		2	15		14	3		5	12	
WBC												
<10,000/mm^3^	10	27	NS	16	21	NS	26	11	NS	11	26	NS
≥10,000/mm^3^	9	8		6	11		15	2		5	12	
Prognostic Index												
0, 1	6	15	NS	12	9	NS	13	8	NS	6	15	NS
2, 3, 4	13	20		10	23		28	5		10	23	

* The American Joint Committee on Cancer tumor-node-metastasis staging system, 8th edition; NS, not significant. WBC, White blood cell count.

## Data Availability

The data presented in this study are available on request from the corresponding author.
